# The impact of nurse working hours on patient safety culture: a cross-national survey including Japan, the United States and Chinese Taiwan using the Hospital Survey on Patient Safety Culture

**DOI:** 10.1186/1472-6963-13-394

**Published:** 2013-10-07

**Authors:** Yinghui Wu, Shigeru Fujita, Kanako Seto, Shinya Ito, Kunichika Matsumoto, Chiu-Chin Huang, Tomonori Hasegawa

**Affiliations:** 1 Division of Health Policy & Health Service Research, Department of Social Medicine, Toho University School of Medicine, 5-21-16 Omori-Nishi, Ota-ku, Tokyo 143-8540, Japan; 2 Department of Senior Citizen Service Management, Ming-Hsin University of Science and Technology, Hsinchu, Taiwan

**Keywords:** Patient safety, Patient safety culture, Nurse working hours, Adverse events

## Abstract

**Background:**

A positive patient safety culture (PSC) is one of the most critical components to improve healthcare quality and safety. The Hospital Survey on Patient Safety Culture (HSOPS), developed by the US Agency for Healthcare Research and Quality, has been used to assess PSC in 31 countries. However, little is known about the impact of nurse working hours on PSC. We hypothesized that long nurse working hours would deteriorate PSC, and that the deterioration patterns would vary between countries. Moreover, the common trends observed in Japan, the US and Chinese Taiwan may be useful to improve PSC in other countries. The purpose of this study was to clarify the impact of long nurse working hours on PSC in Japan, the US, and Chinese Taiwan using HSOPS.

**Methods:**

The HSOPS questionnaire measures 12 sub-dimensions of PSC, with higher scores indicating a more positive PSC. Odds ratios (ORs) were calculated using a generalized linear mixed model to evaluate the impact of working hours on PSC outcome measures (patient safety grade and number of events reported). Tukey’s test and Cohen’s d values were used to verify the relationships between nurse working hours and the 12 sub-dimensions of PSC.

**Results:**

Nurses working ≥60 h/week in Japan and the US had a significantly lower OR for patient safety grade than those working <40 h/week. In the three countries, nurses working ≥40 h/week had a significantly higher OR for the number of events reported. The mean score on ‘staffing’ was significantly lower in the ≥60-h group than in the <40-h group in all the three countries. The mean score for ‘teamwork within units’ was significantly lower in the ≥60-h group than in the <40-h group in Japan and Chinese Taiwan.

**Conclusions:**

Patient safety grade deteriorated and the number of events reported increased with long working hours. Among the 12 sub-dimensions of PSC, long working hours had an impact on ‘staffing’ and ‘teamwork within units’ in Japan, the US and Chinese Taiwan.

## Background

In 1999, the US Institute of Medicine reported that 44,000–98,000 inpatients in US hospitals are estimated to die because of adverse events each year [1]. Another report mentioned that approximately 1.3 million patients are injured by adverse events during their hospitalization each year [2]. Patient safety is of greatest concern throughout the world. A positive patient safety culture is one of the most critical components that could improve quality and safety in healthcare [3].

Patient safety culture (PSC) is defined as ‘the product of individual and group values, attitudes, perceptions, competencies and patterns of behaviour that determines the commitment to, and the style and proficiency of, an organization’s health and safety management’ [4]. The Hospital Survey on Patient Safety Culture (HSOPS), developed by the US Agency for Healthcare Research and Quality (AHRQ), has been used to assess PSC in 31 countries including Japan and Chinese Taiwan (Taiwan) [5-8].

Professional staff nurses are most likely involved in adverse events and an estimated 11 million nurses throughout the world have a critical impact on safety initiatives [9]. Several studies have indicated that long working hours have adverse effects on nurse performance. Linda *et al.* showed that nurses working >40 h/week had an increased chance to make both errors and near misses [10]. Rogers *et al.* found that the risks of making an error increased significantly when work shifts were >12 h, or when staff worked for >40 h/week [11]. These studies were based on voluntary reports of errors or near misses. As the increase in reported events can be interpreted as deterioration in patient safety as well as an improved PSC, which enables better detection and reporting, it might be difficult to determine the impact of long working hours on patient safety using the number of reports as an indicator. An assessment of PSC might be an alternative method to evaluate the impact of long working hours on patient safety. The impact of long working hours on PSC might vary between countries because PSC cultural differences have been identified among healthcare workers in Japan, Taiwan and the US [8]. We hypothesized that long working hours would deteriorate PSC, and that the deterioration patterns would differ between countries. Moreover, the common trends observed in Japan, the US and Taiwan may be useful to improve PSC in other countries.

The purpose of this study was to clarify the impact of long working hours on PSC in countries with different cultural backgrounds using the nurse PSC databases in Japan, the US and Taiwan.

## Methods

### Data sources and study design

We obtained the US and Taiwan HSOPS data from US AHRQ and a research team in Taiwan, respectively. We also conducted a cross-sectional anonymous survey and constructed a Japanese HSOPS database. The characteristics of the participating hospitals in the three countries are shown in our previous study [8]. Only nurse-related data from the above-mentioned databases were used, because they were the target respondents of this study. Incomplete questionnaires, such as surveys with less than an entire section completed, those with fewer than half the total items completed, or those with identical responses to every item, were excluded from our analysis [5,6].

The US data were a 2010 HSOPS database collected from 884 hospitals from January 2006 to June 2009. All hospital staff at 14 hospitals in Japan were asked to complete the Japanese HSOPS from January 2009 to January 2010. The Taiwanese HSOPS was distributed to a part of the hospital staff in 74 hospitals in Taiwan, from July 2007 to August 2008, chosen from all 556 hospitals in Taiwan by stratified sampling with proportional allocation. The details of the data are shown in our previous study [8].

### Questionnaire

HSOPS has 51 questions divided into three parts as follows: background information (seven items), outcome measures of PSC (two items) and sub-dimensions of PSC (42 items). The background information section includes items regarding respondent’s primary unit, staff position, working hours per week and years in current specialty. Outcome measures in the PSC section have two items: (a) patient safety grade, which is measured using a five-point Likert scale from ‘excellent’ to ‘failing’ for rating patient safety in the respondent’s workplace; (b) number of events reported by the respondent during the past year. Sub-dimensions of the PSC section include 42 items with a five-point Likert scale of agreement (from ‘strongly disagree’ to ‘strongly agree’) or frequency (from ‘never’ to ‘always’). These 42 items are categorized into the following categories: (1) frequency of event reporting, (2) overall perceptions of safety, (3) supervisor/manager expectations and actions promoting safety, (4) organizational learning-continuous improvement, (5) teamwork within units, (6) communication openness, (7) feedback and communication about errors, (8) nonpunitive response to error, (9) staffing, (10) hospital management support for patient safety, (11) teamwork across hospital units and (12) hospital handoffs and transitions. Among these sub-dimensions, ‘staffing’ was defined as the number of staff required to handle the workload and work hours appropriate to provide the best care for patients. ‘Teamwork within units’ was defined as the level at which staff support one another, treat one another with respect and work together as a team [5].

Internal reliability and construct validity of the questionnaire were verified in previous studies [6-8].

### Data analyses

First, the 42 items in the sub-dimensions of PSC were scored on a five-point Likert scale and scores for negatively worded items were reversed. AHRQ recommends using percentages of positive answers for each sub-dimension, but those calculation methods decrease the amount of information during numerical transformation. Therefore, in this study, responses to each item within the same sub-dimension were summed, and the mean of each PSC sub-dimension was calculated, resulting in a sub-dimension score of 3–15 points if there were three items in the sub-dimension.

Second, to estimate the effect of working hours on patient safety grade and number of events reported during the past year, the odds ratio (OR) of working hours per week for patient safety grade and number of events reported were calculated using a generalized linear mixed model (GLMM). The ORs showed the possibility to have high patient safety grade, or to have experience of event reporting. The differences between each hospital and years in their current specialty or profession were included in the GLMM as random effects for measuring the effect of working hours on patient safety grade and number of events reported because patient safety grade and number of events reported were affected by working hours, years in their current specialty or profession and the hospitals where the respondents work.

Finally, to measure the effects of working hours on sub-dimensions of PSC, nurses were divided into three groups by working hours per week (<40 h; 40–60 h; ≥60 h), and we used Tukey’s test to verify whether there were differences in each sub-dimension of PSC among different working-hour groups. To confirm whether the difference was substantially meaningful or not, Cohen’s *d* values were used to indicate the effect size for the mean differences in the 12 sub-dimensions among the working-hour groups. Cohen described a *d* value of 0.2 as being small, 0.5 as medium and 0.8 as large effects [12].

Chi-square tests were used to compare categorical variables. Descriptive statistics, GLMM, Tukey’s tests and chi-square tests were performed with SPSS Statistics ver. 19.0 (SPSS, Inc., Chicago, IL, USA). Cohen’s *d* values were calculated using the open-source R software, version 2.12.1.

### Ethical concerns

According to the Ethical Guidelines for Epidemiological Research, which was drawn up by the Japanese Ministry of Health, Labor and Welfare, approval of the ethics committee was not required because this study was an anonymous and self-administered survey with no intervention or mental anguish [13]. The survey was approved in Taiwan by the Institutional Human Subject Ethic Committee of National Chung Cheng University.

## Results

The US 2010 HSOPS database consisted of 337,862 respondents and the response rate was 41.0%. Among respondents, 114,818 (34.0%) were nurses. After incomplete questionnaires were excluded, 106,710 (37.0%) nurses were included. In Japan, 8,192 respondents completed questionnaires and the response rate was 66.5%. Among the respondents, 4,255 (52.0%) were nurses including 4,047 (58.1%) valid data. In Taiwan, 10,289 hospital staff completed questionnaires, and the response rate was 88.0%. Among the respondents, 5,773 (56.1%) were nurses, in which valid data were available for 5,714 (56.3%).

The respondent’s characteristics are shown in Table 1. US nurses were more likely to rate patient safety conditions of their work area as ‘excellent’ or ‘very good’ compared with Taiwan (P < 0.001) and Japan (P < 0.001). The proportion of nurses in Taiwan reporting no events during the past year was higher than that in the US (P < 0.001) and Japan (P < 0.001).

**Table 1:** Respondent characteristics

			**Japan**		**U.S.**		**Taiwan**
		***n***	**%**	***n***	**%**	***n***	**%**
Working hours per week in this hospital	<40 h	930	23.0	61,904	58.0	936	16.4
	40–60	2,352	58.1	35,488	33.3	4,226	74.0
	≥60 h	217	5.4	5,656	5.3	466	8.2
	No answer	548	13.5	3,662	3.4	86	1.5
Years in current specialty	<1	335	8.3	6,033	5.7	491	8.6
	1–5	1,329	32.8	25,339	23.7	2,355	41.2
	6–10	846	20.9	17,261	16.2	1,521	26.6
	11–15	541	13.4	13,889	13.0	740	13.0
	16–20	319	7.9	12,195	11.4	345	6.0
	≥21	535	13.2	27,704	26.0	236	4.1
	No answer	142	3.5	4,289	4.0	26	0.5
Patient Safety Grade	Excellent	135	3.3	22,410	21.0	214	3.7
	Very good	1,662	41.1	48,325	45.3	1,791	31.3
	Acceptable	1,740	43.0	25,948	24.3	2,807	49.1
	Poor	282	7.0	6,147	5.8	223	3.9
	Failing	36	0.9	990	0.9	22	0.4
	No answer	192	4.7	2,890	2.7	657	11.5
Number of Events Reported (during the past 1 year)	No event reports	717	17.7	31,506	29.5	2,523	44.2
	1–2	1,759	43.5	42,401	39.7	2,159	37.8
	3–5	1,145	28.3	21,555	20.2	649	11.4
	6–10	317	7.8	6,631	6.2	163	2.9
	11–20	58	1.4	2,044	1.9	45	0.8
	≥21	17	0.4	898	0.8	23	0.4
	No answer	34	0.8	1,675	1.6	152	2.7
Total		4,047	100.0	106,710	100.0	5,714	100.0

The effects of working hours on outcome measures of PSC are shown in Table 2. Compared with nurses working <40 h/week, nurses working ≥60 h/week gave patient safety grade a poorer rating (Japan: OR, 0.65; US: OR, 0.82; Taiwan: OR, 0.84) and had more event reporting experiences (Japan: OR, 2.74; US: OR, 1.11; Taiwan: OR, 1.85).

**Table 2:** Effect of nurse working hours on patient safety grade and number of adverse events reported

	**Working hours per week**	**P value**	**Odds ratio**	**95% CI**
Patient safety grade				
Japan	<40		1.00	
	40–60	0.31	0.92	0.78–1.08
	≥60	0.01^*^	0.65	0.46–0.90
U.S.	<40		1.00	
	40–60	0.11	1.03	0.99–1.06
	≥60	<0.001^**^	0.82	0.78–0.88
Taiwan	<40		1.00	
	40–60	0.47	1.06	0.90–1.26
	≥60	0.18	0.84	0.64–1.09
Number of events reported				
Japan	<40		1.00	
	40–60	<0.001^**^	1.72	1.41–2.09
	≥60	<0.001^**^	2.74	1.68–4.47
U.S.	<40		1.00	
	40–60	<0.001^**^	1.08	1.05–1.11
	≥60	0.002^**^	1.11	1.04–1.18
Taiwan	<40		1.00	
	40–60	0.009^**^	1.23	1.05–1.43
	≥60	<0.001^**^	1.85	1.45–2.36

*P < 0.05, **P < 0.01; CI, confidence interval.

The effects of working hours on each sub-dimension of PSC are shown in Table 3 and the Additional file 1. In regard to ‘staffing’ and ‘teamwork within units’, the mean scores of nurses who worked ≥60 h were significantly lower than the mean scores of nurses who worked <40 h if the effect size was considered (‘staffing’; Japan: P < 0.001, *d* = 0.50, US: P < 0.001, *d* = 0.20, Taiwan: P < 0.001, *d* = 0.60, ‘teamwork within units’; Japan: P = 0.02, *d* = 0.27, US: P < 0.001, *d* = 0.14, Taiwan: P = 0.005, *d* = 0.24).

**Table 3:** Mean scores of each sub-dimension in the different working hour groups

	**Japan**			**U.S.**			**Taiwan**		
	**(*n *= 4,047)**			**(*n *= 106,710)**			**(*n *= 5,714)**		
**Sub-dimensions**	**<40 h**	**40–60 h**	**≥60 h**	**<40 h**	**40–60h**	**≥60 h**	**<40 h**	**40–60 h**	**≥60 h**
	**(*n *= 930)**	**(*n *=2,352)**	**(*n *= 217)**	**(*n *= 61,904)**	**(*n *= 35,488)**	**(*n *= 5,656)**	**(*n *= 936)**	**( *n *= 4,226)**	**(*n *= 466)**
Frequency of Event Reporting	12.4	12.5	12.3	11.0	11.2	11.2	9.3	9.3	9.4
Overall Perceptions of Safety	14.0	13.7	13.5	13.8	13.9	13.5	13.2	13.4	13.1
Supervisor/Manager Expectations & Actions Promoting Safety	15.0	14.8	14.8	15.2	15.3	14.9	14.4	14.7	14.2
Organizational Learning-Continuous Improvement	10.7	10.6	10.6	11.2	11.3	11.2	11.6	11.7	11.5
**Teamwork within Hospital Units**	**15.3**	15.1	14.6*	**15.8**	15.7	**15.4**	**15.5**	15.5	14.9*
Communication Openness	10.6	10.5	10.4	10.9	11.0	10.7*	9.8	9.8	9.2*
Feedback and Communication about Error	11.	11.1	11.0	10.8	11.0	10.9	10.1	10.2	9.9
Nonpunitive Response–Error	9.7	9.6	9.4	9.4	9.5	9.0	8.6	8.7	8.0*
**Staffing**	**12.2**	11.8	10.9*	**13.7**	13.5	13.1*	**12.0**	11.9	10.6*
Hospital Management Support for Patient Safety	10.4	10.3	10.2	10.7	10.8	10.6	10.6	10.6	10.2*
Teamwork Across Hospital Units	13.2	13.0	12.9	13.4	13.3	13.2	13.6	13.7	13.1*
Hospital Handoffs & Transitions	12.7	12.5	12.2	12.8	12.	12.2	12.8	12.7	12.3

Each dimension is rated from 1–5 points and has three or four items resulting in a mean score of a dimension either from 3–15 points or 4–20 points; *SD* standard deviation.

*Cohen’s *d* ≥ 0.2 and *P* < 0.05, compared with the <40-h group.

## Discussion

The results of this study suggest that the long working hours of nurses deteriorated the patient safety grade of their work area and increased the number of nurses with event reporting experience in the three countries. The ratings for ‘staffing’ and ‘teamwork within units’ deteriorated as a result of long working hours.

In the US, more than half of nurses worked <40 h/week in contrast to Japan and Taiwan. In US hospitals, temporary nurses, who are identified as ‘agency nurses’ or ‘travel nurses’ are popular, although most hired nurses in Japan and Taiwan are permanent staff.

Nurses who work fewer hours were more likely to rate patient safety condition of their work area as ‘excellent’ or ‘very good’. Nurses who worked <40 h/week might be part-time workers who undertook uncomplicated tasks, whereas other nurses might have an increased chance not only to make but also to detect errors or near misses due to long working hours. The relationship between errors and long working hours has been reported previously [10,11,14,15]. Rogers *et al*. reported that nurses working >12.5-h shift or working for ≥12.5 h in a 24-h period had 3.29 times increased likelihood of making an error (OR, 3.29, p = 0.01) compared with the 8.5-h shift group [11]. In contrast, the increased number of reports could be considered a sign of positive PSC under which the staff easily detected errors and felt free to report. In our study, long working hours were related to poor PSC and an increase in the number of adverse events reported. An analysis adjusted for working hours or work stress might be needed to measure the relationship between a good PSC and the increase in the number of reported events.

In all the three countries, when nurses worked ≥60 h/week, the sub-dimension ‘staffing’ was rated poorer than those who worked <40 h/week. Previous studies reported similar results, although they did not use HSOPS [16-20]. ‘Nurse to patient ratio’, ‘time of nursing care’, ‘nurse full time equivalents’ and ‘nurse whole time equivalents’ were used as surrogate indicators of staffing. Long working hours might be related to poor staffing. If nurses needed to work ≥60 h/week, there might not be sufficient staff to handle the workload and they might be forced to work in a ‘crisis mode’; trying to do too much too quickly.

In Japan and Taiwan, the situation of ‘teamwork within units’ in nurses working <40 h/week was better than that of nurses working ≥60 h/week. Kalisch *et al.* in 2009 found that nurses working <30 h/week rated their teamwork score significantly higher than those working >30 h/week [21]. Other studies have reported that fatigue and stress scores in nurses who work a 12-h shift are significantly higher than those of nurses who work an 8-h shift [22,23]. Thus, increased fatigue, work intensity or work stress caused by long working hours might lead to mistakes or communication and interpersonal problems resulting in less teamwork without respect, understanding, support and helping one another [11,21-23]. Hospital managers may want to coordinate nurse working schedules to keep working hours appropriate to establish a good PSC. Such efforts might lead to a decrease in the number of adverse events due to miscommunication.

Although the two sub-dimensions of ‘staffing’ and ‘teamwork within units’ were related to working hours, the common deterioration patterns due to long working hours were not identified in the other 10 sub-dimensions. The difference in the deterioration pattern between countries might result from different mechanisms reflecting the cultural backgrounds.

This study had some limitations. It was unclear how the actual workload or work intensity affected PSC because objective indicators of ‘staffing’ such as patient–nurse ratio or patients’ severity were not collected. Because of the sampling method in each country, the target population might not be representative of the entire country. The US response rate was lower than that of Japan and Taiwan, and the characteristics of non-respondents were unknown.

## Conclusions

Patient safety grade deteriorated and the number of events reported increased with longer working hours among nurses in the three countries. Among 12 sub-dimensions of PSC, only the two sub-dimensions of ‘staffing’ and ‘teamwork within units’ were rated poorer in common when nurses worked long hours.

## Abbreviations

HSOPS: Hospital survey on patient safety culture; PSC: Patient safety culture; AHRQ: Agency for healthcare research and quality; OR: Odds ratio; GLMM: Generalized linear mixed model.

## Competing interests

The authors declare that they have no competing interests.

## Authors’ contributions

YW performed the statistical analysis and wrote the manuscript. SI and SF participated in the design of the study and performed the statistical analysis. CH, KM, KS, SI and SF collected the data and revised the manuscript. TH managed the entire research process. All authors read and approved the final manuscript.

## Pre-publication history

The pre-publication history for this paper can be accessed here:

http://www.biomedcentral.com/1472-6963/13/394/prepub

## Supplementary Material

Additional file 1 The difference among working hour groups by Tukey’s test in Japan, the U.S. and Taiwan. Click here for file
